# The risk of normative bias in reporting empirical research: lessons learned from prenatal screening studies about the prominence of acknowledged limitations

**DOI:** 10.1007/s11017-023-09639-x

**Published:** 2023-11-06

**Authors:** Panagiota Nakou, Rebecca Bennett

**Affiliations:** Department of Law, Centre for Social Ethics and Policy, The University of Manchester, Manchester, M13 9PL UK

**Keywords:** Empirical bioethics, Policy, Prenatal screening, Bias, Reflexivity, Data

## Abstract

Empirical data can be an extremely powerful and influential tool in bioethical research. However, when researchers or policy makers look for answers to ethical questions by engaging with empirical research, there can be a tendency (conscious or unconscious) to shape, report, and use empirical research in a way that confirms their own preferred ethical conclusions. This skewing effect - what we call ‘normative bias’ - is often so subtle it falls short of clear misconduct and thus can be difficult to call out. However, we argue that this subtle influence of bias has the potential to significantly influence debate and policy around highly sensitive ethical issues and must be guarded against. In this paper we share the lessons we have learned through a journey of self-reflection around the effect that normative bias can have when reporting on and referring to empirical data relating to ethical issues. We use a variety of papers from our area of the ethics of routine prenatal screening to illustrate these subtle but often powerfully distorting effects of bias. Our aim in doing so is not to criticise the work of others, as we recognise our own normative bias, but to improve awareness of this issue, remind the need for reflexivity to guard against our own biases, and introduce a new criterion - the idea of a ‘limitation prominence assessment’ - that can work as a practical way to evaluate the seriousness of the limitations of an empirical study and thus, the risks of the study being misread or misinterpreted through superficial reading.

## Introduction

Empirical research and the data it generates can be an extremely powerful and influential tool in bioethical research. Although as researchers we approach bioethics from the traditional school of the methodology of philosophical reasoning and inquiry, we acknowledge the fundamental role of empirical research in bioethics, particularly when it comes to recommendations for policy around ethically sensitive issues. This is markedly apparent in our own area of research around the ethics of routine prenatal screening, where research data on women’s opinions about using this technology powerfully informs the ethical debate on whether screening enhances the autonomy of their choices or curtails women’s reproductive choices.^1^ Given the often pivotal role of empirical data when attempting to come to a conclusion on bioethical issues, particularly when it comes to decisions on policy, it is clearly paramount to ensure that the data is generated, reported, and used accurately. There is a wealth of literature emphasising the responsibility of bioethicists in the way that they engage with empirical data [1–5]. For instance, there is evidence that many consumers of empirical data, particularly those relating to issues of ethical relevance, may not look critically at the way that empirical data are presented before using it [1, 6] and that there can be a tendency to “cherry pick” information from empirical studies selectively to endorse one’s own normative claims [6]. While this gives reason to urge these consumers to take more care and be more critical and reflexive in the way that they use empirical data, it also, we argue, puts the onus on researchers presenting empirical research to do so with *extra* responsibility.

Using examples from our own area of research—the ethics of routine prenatal screening—we will show how the normative claims of those generating empirical data may influence, however subtlety, the way that this empirical data is presented. This effect, resulting from what we call “normative bias” on the way that empirical work can be reported, is subtle and usually falls short of what could be considered malpractice. However, it has the potential to significantly influence the ethical debate particularly when one recognises the often uncritical use of this data. Our aim in this paper is not to criticise the work of others, as we recognise our own normative bias and the potential influence this has on our own work. The aim of this paper, rather, is to shine a light on this issue and to argue that this subtle influence of normative bias places a strong obligation on researchers — if they wish to maintain the integrity of the work they present — to be *extra* vigilant and *extra* cautious when reporting empirical projects that have relevance to ethical issues. Also, because realising and controlling our implicit biases is very difficult and often impossible, we suggest that a secondary failsafe should be put in place by publishers in order to help authors to present their research in ways that are more resistant to secondary misuse. Accordingly, we suggest criteria that can contribute in this direction under what we term a “Limitation prominence assessment”.

## The intersection between bioethics, empirical research, and policy

While there will always be debate about what healthcare policies should exist in a particular area, it is uncontroversial that good or defendable policy should attempt to reflect what is ethically acceptable. Indeed, deciding what is ethically acceptable in many areas of healthcare is challenging. Many ethical questions — such as whether it is acceptable to destroy a human embryo or whether parents should be allowed to select the sex of their children — lead to not just polarised debates, but splintered debates with almost as many different positions on these questions as there are individuals talking about these issues. This is, of course, highly problematic for policy makers as there is no consensus on the right thing to do. Further, while it may be possible to base policy on a compromise position in some areas of regulation (e.g., the division of property), finding a true compromise position on ethical questions is often impossible as — what is often billed as compromise — usually involves one side losing a great deal to the other (e.g., the United Kingdom’s 14-day rule on the use of human embryos).

And yet there is the need for policy in areas of ethical controversy, particularly those relating to the provision of healthcare. As a result, there has been a tendency for healthcare policy to be driven chiefly by technical and scientific discoveries [7]. There may also be attempts to find more “scientific” solutions to the ethical problems facing policy makers; in particular, consider the use of public consultation as a way of attempting to move forward in the face of a significant lack of consensus regarding ethical issues [8].

While finding out what the public thinks about an ethical issue is important, we know from experience that what the public thinks does not always align with what may be ethically justified. In the past, there has been widespread public support for criminalising homosexuality and for policies that enforced gender and racial inequality [9–12], positions that are difficult to defend ethically. Thus, asking the public what they think about an ethical issue will only reflect what those asked think about this issue, and may not yield insight into what is the most ethically justifiable policy response in a given area. As a result, for many ethical questions, policy makers are the ones left to decide what the most defendable position is by weighing the different sides of the argument in a similar way done within philosophical bioethics.

Arguably, however, there are some ethical questions that may be answered — or at least significantly informed by — scientific rather than philosophical methodology. Considering our own area of research — the ethics of routine prenatal screening — we argue that, unlike many other areas of ethical controversy, asking individuals what they think about routine prenatal screening *does* have the potential to be highly illuminating when it comes to developing ethically defendable policy in this area.

The issue of Non-Invasive Prenatal Testing (NIPT) has recently refocused and reignited the debate around routine prenatal screening, most notably around the use of screening to identify conditions such as Down Syndrome. This debate around routine prenatal screening is polarised, with those on one side claiming that screening is a means to empower women and enhance their reproductive autonomy [13, 14, p. 743], while those on the other side argue that screening has eugenic aims and puts pressure on women to terminate pregnancies that, given a freer choice, they may not have terminated [15–18]. Given that this ethical debate essentially pivots on whether these kind of screening programmes do in fact empower women or curtail their choices, any empirical data that seeks to illuminate the reality of women’s experience in these situations will be fundamental in understanding the impact of this screening on women’s choices.

## The important role of empirical research in bioethics and the responsibility that comes with it

There is a well-established body of literature that explores the importance [19–22] of empirical research in bioethics. It is acknowledged that empirical research and the data it generates can make ‘bioethics more effective’ [21, p. 41] by connecting the conception of the idea of a better world with the actual world [4, 21]. While there will be those who argue that empirical research data cannot determine what is ethically right or wrong [4, 23], it may also be argued that ‘ethical theory, ethical norms, and values are nurtured and shaped by empirical knowledge’ [4, 23, p. 71]. As a result, empirical research is often integrated into the bioethical debate. Furthermore, empirical data is often seen as something much more objective, convincing, and “evidence based” than more traditional philosophical bioethical argument, reflection, and debate. As a result, empirical data can be an extremely powerful tool in this debate, providing insight into the reality of women’s experience in a way that will be seen as objective, compelling, and having the potential to be highly attractive and valuable to those engaging in policy making around prenatal screening. However, while empirical research has a highly valuable and perhaps even pivotable role to play in this ethical debate, this fundamental role of empirical research comes with some serious challenges that need to be addressed for this role to maintain integrity.

## Research integrity

Research misconduct is a long-standing and international problem in empirical research with equally long-standing efforts to deal with this problem. For instance, Soehartono and Khor inform us that ‘between 1990 and 2020, over 9700 publications were published to address problematic research conduct such as falsification, plagiarism, and related protocols and standards’ [24, p. 7487].

Although there is no consensus on the definition of research misconduct, fabrication, falsification, and plagiarism are generally acknowledged as practices of misconduct [25, pp. 252–253]. Additionally, the reasons and incentives behind research misconduct have been explored. Among others, research misconduct has been attributed to the researchers’ ambition, incrementalism, group, and authority pressure [26] as well as publication pressure [27, p. 375]. Namely, it has been argued that given the differences in the way researchers approach the representation and interpretation of results, ‘they may be tempted consciously or unconsciously to shape the impression that the results will have on readers and consequently “spin” their study results’ [28, p. 2613]. Boutron and Ravaud define spinning as ‘a specific intentional or unintentional reporting that fails to faithfully reflect the nature and range of findings and could affect the impression the results produce in readers’ [28, p. 2613]. The argument here is that researchers, like all human beings, view the world around them, including their research, through their own lens of their conscious and unconscious biases. In the reporting of empirical research, this spinning might take various forms—from misreporting methods or results, to misinterpretation of results, or to more subtle types of spin—where the way that the results are presented and the language used might be seen as encouraging a reading of these results that produces an impression of the research that cannot actually be supported by the research results. According to Chan et al. [29] ‘[p]ublication bias and outcome reporting bias are two of the main issues regarding research communication misbehaviors’ [30, p. 4]. Such practices are likely to support the agendas of authors, but like most human biases it may well be that the author remains unaware of the biases they have and the effect these biases have on their reporting.

### Bioethics, empirical data, intuition, and bias

Bioethicists engaging with empirical studies and data must guard against bias and spin when presenting their research to maintain high standards of research integrity. A number of commentators have pointed out pitfalls [1–5] that need to be addressed when it comes to the use of data in bioethics which focus predominantly on accurate assessment of the validity of the data and the misuse of secondary data. These pitfalls include papers where the conclusions reached were not linked to the cited data or where ‘strong (and general) conclusions’ [1, p. 68] were based on limited data of instances where authors uncritically and wrongly linked empirical claims with the cited data [1, p. 71]. On the issue of misusing secondary data, it is not uncommon for those who use empirical data to inform their ethical arguments, if not vigilant, to fall into the trap of (consciously or unconsciously) selecting or representing data so that empirical results are used to confirm their own ethical take on an issue, something we refer to here as “normative bias” [3]. While reason and logic have been considered pillars of objectivity among philosophical bioethicists aiming to protect their view of the truth from subjective thinking [6], it has been argued that ethical arguments are invariably susceptible to being shaped by intuition [6, 31–33].^2^ Ives and Dunn, for instance, argue that:

…moral argument becomes something of a servant to the master that is our moral intuition – inciting us to act only when it finds an existing sympathy and only surfacing to reinforce and justify a conclusion already formed by our intuitions [6, p. 259].

Accordingly, when it comes to using empirical research data as part of ethical analysis, there may be a temptation (conscious or unconscious) to cherry-pick or misuse such data to reinforce and justify an argument or conclusion that satisfies the author’s moral intuition [6]. This is possibly worsened by the fact that many of the researchers engaging with empirical data on ethical issues may not have a classical social science background, and, as a result, might not be as aware of what it means to engage critically and comprehensively with the literature. However, this wealth of literature, once engaged with, illuminates the importance of these issues for those engaging with empirical research in bioethics.

Sugarman and Sulmasy, for example, have highlighted the importance of recognizing this validity for those who consider empirical data ‘to inform their conceptual research in bioethics’ [3, 19, p. 67; ]. By this, they mean that it is paramount that bioethicists — and others working in the area of ethics — acknowledge that the quality of empirical research varies [3, pp. 66–67] and that one should be critical when choosing which empirical data to consider in one’s ethical analysis. Sugarman et al. explain further, arguing that authors using empirical data should be cautious as ‘to how conclusive individual studies are, and when multiple studies are necessary to inform one’s purposes’ [3, p. 67]. Similarly, Provoost suggests it is important for authors to reflect ‘on the quality of the data they are using (as a basis of argumentation) in order to guarantee that they “abide by a high threshold of evidence”’ [1, 3, p. 73; ].

### A framework for vigilance

These lessons are well established in the literature around empirical bioethics. In addition to these lessons, it has been suggested that a widely acceptable methodological approach capable of integrating ‘the empirical and the normative part of empirical ethics’ [34, p. 1] would be helpful in establishing particular standards to assess the quality of empirical ethics research work. While there have been suggestions on methodologies which can be used to support integrity in empirical bioethics research [35–41], as Davies et al. observed, there is still ‘no standard approach to cite, there is no accepted methodology or set of methods to fall back on, and the process of offering justification for every methodological choice from first principles takes a lot of space, which is rarely available’ [42, p. 12]. This can lead to objective difficulties in achieving the best quality in this type of work. For instance, in considering the common issue of poor reporting of results in empirical ethics research, Frith and Draper explain that:

this type of research does not have its own established reporting norms and has to fit in with either the norms of bioethics and philosophical-style papers or the requirements for empirical papers (such as those that require papers to be structured with background, methods, results and discussion). Adopting either approach will involve compromises in the reporting of the data [43, p. 20].

Trying to combine reporting empirical data with the related ethical reasoning in research ethics papers can be a challenging task given the restrictions put by journals regarding the format and word limits. For instance, in cases of tight word limits, authors can be faced with a choice of either providing a rigorous analysis of the research data and method or analysing the ethical implications of the reported data. Either way, the quality of the paper is negatively affected in such cases [43, p. 20].

According to the above, we do understand that there are practical difficulties that can limit the quality of research ethics papers and therefore, researchers are sometimes forced to make compromises. However, we argue that there are still actions that researchers can take to guard the quality and integrity of their work which can be mutually agree upon. It is, of course, important to recognise and reiterate the points made by others to encourage bioethicists, researchers from different fields, and policy makers — essentially anybody aiming to provide an answer to an ethical question by picking up or doing empirical research — to examine the way they engage with empirical research and do so critically and with self-awareness of the often unconscious influence of normative bias. However, in this paper, we suggest that there is one particular check that could be instigated now, which we hope would enable increased vigilance within empirical ethics and, perhaps in time, become one element in a framework for encouraging research integrity unilaterally. In the rest of this paper, we outline the particular concerns that led to this recommendation and the recommendation itself, something we call the “Limitation prominence assessment”.

## The background to our recommendation

As bioethical researchers who raise questions about the quality of consent to routine screening in pregnancy, we often come across empirical research papers focusing on women’s opinions about the offer, use, and ethics of prenatal screening technologies. Such papers can yield valuable insights into the views of women regarding prenatal screening; particularly regarding newly introduced technologies in this field such as NIPT. Given that our work focuses on challenging the way that antenatal screening is often justified as a means of empowering women — when the reality, we argue, is that this screening often presents a challenge to women’s autonomy [16, 44, 45] — we feel that quite often we tend to be more critical and cautious with those papers that do not seem to agree with our point of view (for instance, papers showing general support on the part of women for extended use of NIPT). Our main concern is related to the fact that journalists, policy makers, and others could pick up such research and report significant support for NIPT among pregnant women. This message is one that might calm nervousness from those planning to add NIPT to existing screening programmes targeting all pregnant women and might be used as evidence of support for this new test against the well-publicised concerns of campaign groups such as ‘Don’t Screen Us Out’ [46] and others who raise concerns about increased screening in pregnancy or the routine nature of increased screening. Given our position on this debate, we are very interested to understand such potentially influential conclusions further.

From our point of view, sometimes we find the way that authors represent their data problematic. Although in these papers there may not be a problem with the methodology or the reporting/representation of the results, sometimes we feel that, rather than presenting the data in a clear and objective manner, the papers may encourage a reading of the results that appears to confirm the normative biases of the researchers undertaking this research. We have a number of concerns about the possible role of implicit bias when it comes to presenting data in a way that seems to confirm one’s preferred ethical conclusion. For instance, we can imagine a scenario in which an interesting snapshot of the attitudes of a small group of self-selecting women is presented in a way that has the potential to influence the debate in this area unjustifiably. We are, of course, making assumptions here about the normative biases of authors, but there seems to be good reason to think that there may be unconscious biases toward recommending extensive use of NIPT from some authors. Below, we analyse a few papers to demonstrate this possibility.

These were the thoughts that led us to write this perhaps peculiar paper. While we had a strong sense that something was not quite right with some papers, the subtly of the phenomenon, and the lack of a clear sense of misrepresentation of data, coupled with the recognition that our own normative bias may be affecting how seriously we perceived these issues to be, compelled us to investigate this issue further. This paper does not aim to be a systematic review of the literature or be critical of the work of others but rather offers a recommendation that may improve research integrity and accuracy in empirical bioethics by a) exploring in detail examples that we have found problematic and b) examining our own practice and the possible effect of our own normative bias on how we view these examples.

Our aim was to discern whether our concerns were guided entirely by our own bias or were legitimately reasonable. Our consideration of these papers led to the conclusion that there are points of concern that merit highlighting and discussing. Given human nature, the problem might not be ultimately resolved here, but in the effort to contribute substantially and practically, *we suggest that one effective and feasible step is to begin with a simple instruction regarding the clarity and content of titles and abstracts.* To this end, we recommend the implementation of a common rule, what we call the “Limitation prominence assessment”; we suggest that editors, reviewers, and submitting authors should be asked to consider this when assessing papers submitted for publication.

## Illustration of the issue — looking into the detail of specific example papers

Because this work is not a critique of these papers, we do not provide an exhaustive list of all potentially problematic points. Instead, we limit ourselves to examples that can occur when researchers present empirical data that relate to ethical questions and may encourage the less than critical reading of these papers. While there are other issues that we could focus on, the problem we focus on in these papers concerns when fundamental information about the empirical research is not prominent enough in a research paper and thus may facilitate secondary misuse of the paper’s data. We argue that, while not reaching the threshold of any wrongdoing on the part of the researchers, this issue has significant potential to influence the bioethical debate in unjustifiable ways. The papers considered below may give the impression, unless read in some detail, of a much stronger message than can be drawn from the data gained. Again, while we do not question the validity of the statistical methodology used, we argue that this issue is something that researchers contributing to this debate need to take extra care to guard against.

### When fundamental information about the nature of the empirical research is not prominent enough and likely to facilitate secondary misuse of the paper’s data

We begin with the example of a paper by Bowman-Smart et al. [47] that piqued our interest on these issues and then widen our discussion to other examples. Given that our paper is also an illustration of self-reflection, at this point we should note the two reasons why we have chosen this paper by Bowman-Smart et al. to be the starting point of comparison. Firstly, this is a good example of an empirical research paper focusing on women’s opinions about the offer and use of new prenatal screening technologies that we usually look at to inform our own research. Secondly, given that this paper shows general support for the extended use of NIPT, something we have argued against, we recognise the need to reflect on and investigate our own criticisms here to mitigate the chances of our own normative bias being the source of our discomfort.

One of the first things that struck us about the Bowman-Smart et al. paper is that the title and abstract of this paper do not seem to reflect the detail of the information contained in this paper. The title of this paper is ‘"Is it better not to know certain things?”: views of women who have undergone non-invasive testing of its possible future applications’ [47, p. 231]. However, on further reading of the introduction, the reader learns that NIPT is only available in Australia privately and that only Australian women participated in the study [47, p. 231]. From this information, we can conclude that all participants in this study undertook NIPT privately. While this information is contained in the paper, as the authors were candid about the notable weaknesses (selection bias) of their study sample in their limitations paragraph in the discussion [47, p. 237], it is not there in the title or the abstract of this paper and this, we argue, is problematic. The fact that women who took part paid a significant amount of money to undergo this test is essential to understand the findings reported. We argue that if the authors were aware of the lack of rigor often employed by those who make secondary use of these findings, then the authors would have good reason to be extra careful in making this information explicit from the outset; if not in the title of the paper due to space limitations, then certainly in the abstract.

While the women’s views on NIPT and its future applications are, of course, interesting, we argue that the fact that the women who participated in this study have already paid privately for this test is highly significant to the conclusions that can be drawn from the data. All the women in this small sample were women who had actively chosen to undertake this test privately and at a significant personal cost of 449 Australian dollars [47, p. 237]. We would expect a sample of women who have already had this test and elected to pay for it to have a bias for NIPT and would be expected to express a positive view in using it. It is this expected bias that makes it essential to accurately present the data with this qualifying information.

Of course, it is not uncommon for papers not to include this kind of information in their titles or abstracts, and, given we expect the diligent reader to read the whole paper, we are not suggesting that the authors have done something *wrong* in leaving this detail out. However, we suggest that failing to be clear about these important details makes one’s paper more vulnerable to the sort of secondary use that involves “cherry picking” and lack of criticality that have been identified elsewhere in the ethical literature [1]. If reflexivity and pursuance of the truth are viewed as integral to high-quality empirical research, then taking this *extra* care when formulating both titles and abstracts would seem to be important.

In the literature, one can find other examples of papers with similar issues. In our research, we noticed that frequently, authors do not state in the title the participants’ nationality or where the research took place. As an example of this, consider the paper ‘Preferences for prenatal testing among pregnant women, partners and health professionals’ by Lund et al. [48]. Although in this case the authors clearly describe in the abstract that the participants in their research are Danish and were ‘recruited at public hospitals in the Central and North Denmark Regions’ [48, p. 1] there is no indication of this information in the title of the paper. A similar issue with the title can be observed in the paper ‘Women’s views on the moral status of nature in the context of prenatal screening decisions’ by Garcia et al. [49]. The abstract omits the fact that this work resulted from a qualitative sub-study in which only 59 women were interviewed [49, pp. 461–462]. This information provides a clear idea of the findings’ significance and helps the reader discern the extent to which the findings can contribute to the relevant ethical debate. Not indicating this information early in the paper together with stating in the abstract that the ‘findings have significant implications for ethical guidance in debates about the acceptability and boundaries of control of offspring characteristics by prenatal testing’ [49, p. 461] may give, we argue, a false impression of the significance of this work for the ethical debate. When authors present their data in an unclear way without highlighting important points to help the reader understand the significance, quality, and/or limitations of the data, there will be a danger of contributing to mistaken or unrepresentative use of their data.

In order to cross-check the validity of our concerns and to mitigate the impact of our own normative bias, we searched the literature for papers that do include in their titles and abstracts those elements that we think are essential. This search led us to the conclusion that the validity of our concerns and the importance of our suggestion can be confirmed by contrasting the above-mentioned papers with similar research papers, where authors have much more informative and representative titles and abstracts for their papers. For instance, consider the abstract in the paper ‘Feasibility and acceptance of screening for fragile X mutations in low-risk pregnancies’ by Ryynänen et al. [50]. This paper’s abstract noted that ‘From July 1995 until December 1996, a carrier test was offered at the Kuopio City Health Centre free of charge to all pregnant women in the first trimester following counselling given by midwives on fragile X syndrome’ [50, p. 212]. In this short sentence, one can see how clearly the authors indicate where the study took place and if the women who participated had to pay for the testing. Moreover, it includes additional important information which enhances the accuracy of the abstract and consequently, the reader’s understanding.

There are, of course, many other examples of producing clear and representative titles and abstracts. We note a few more here to illustrate how important getting this right can be in terms of the impression a paper may make. For example, the paper ‘Positive Attitudes towards Non-Invasive Prenatal Testing (NIPT) in a Swedish Cohort of 1,003 Pregnant Women’ by Sahlin et al. [51] informs the reader directly in the title about the content of the paper and includes an indication of the study’s findings and the main features of the sample. Likewise, one could add to the list more titles such as: ‘The value of non-invasive prenatal testing: preferences of Canadian pregnant women, their partners, and health professionals regarding NIPT use and access’ by Birko et al. [52], ‘Canadian Pregnant Women's Preferences Regarding NIPT for Down Syndrome: The Information They Want, How They Want to Get It, and With Whom They Want to Discuss It’ by Laberge et al. [53] and ‘Spanish- and English-Speaking Pregnant Women’s Views on cfDNA and Other Prenatal Screening: Practical and Ethical Reflections’ by Floyd et al. [54].

Accordingly, considering these examples and without any intention to understate the importance of the responsibility on the reader’s part to read critically, we argue that it should be incumbent on authors to clearly represent the data not only in the body of the paper but also in the highly visible abstract to avoid secondary misuse of these data. It is easy to see how someone reading only the title and abstract of a paper like Bowman-Smart et al. or Garcia et al. might use this research to illustrate or back up their argument or policy recommendation in a way that a more careful reading of the paper would not allow. Hence, while readers clearly have a responsibility to be critical of what they read, we suggest that there remains a primary responsibility of the authors to protect their work from unwanted misuse by providing a full and accurate description of the research from the outset.

To reiterate the importance of this point we consider another example paper, in which fundamental information about the nature of the empirical research is not prominent enough. The paper is: ‘Screening for fragile X syndrome in women of reproductive age’ by Pesso et al. which focuses on the examination of the rates of carrier identification with extended use of screening for fragile X syndrome in a low-risk population [55]. Although at first sight, this paper does not seem to match with our central focus on views of women about prenatal screening, we have considered it here because it presents important findings of the uptake of prenatal diagnosis from pregnant women who, interestingly, made up the majority of their participants. In detail, 80% of the women who participated were pregnant; further prenatal diagnosis was carried out in a number of ‘concurrent or subsequent pregnancies among carriers’ [55, p. 611]. Moreover, the authors highlight in their conclusions that ‘the uptake of prenatal diagnosis was high’ [55, p. 614]. This, as in the Bowman-Smart et al. paper, is the information about the private type of testing carried out for this research which ‘is only partially covered by health insurance and women have to pay themselves’ [55, p. 611].

As we have discussed above, the fact that the testing in question was privately paid for by the participants in this research is, we argue, a significant limitation likely prone to selection bias. Although Pesso et al. do mention this limitation, this information, we argue, is not prominent enough in the paper. Similar to Bowman-Smart et al., in Pesso et al. the information about this testing as privately funded is not encountered until much later in the paper — in the “materials and methods” [55] section and at the very end of the paper. Furthermore, at the point where the authors note the high uptake of prenatal testing in their study, they do not clarify and highlight that this may be linked to a selection bias as the participants in this study were ones that had sought out this testing and elected to pay for it.

We argue that since the authors decided to consider the significant point regarding the high uptake of prenatal diagnosis, they should have also highlighted the importance of the limitation resulting from the private nature of this testing. We suggest that this information could have been made explicit from the outset. Alternatively, it could have accompanied the information about the high uptake as an explicit clarification to help the reader understand the impact of this limitation on this finding and the real significance of the finding too.

But why does this matter you might say? While these papers might not be as explicit as they could have been, the information about any limitations of the data is there for those who read the paper thoroughly. This may seem like a minor issue to many who may still feel that we are making a great deal out of nothing. In response, we would like to try and illustrate why we argue this is potentially a very significant issue with two examples of how the way information is presented in the papers by Bowman-Smart et al. and Pesso et al. has contributed to secondary misuse of their data which can negatively influence ethical debate.

### Bowman-Smart et al. in Winter

While there will be those who remain sceptical about our concerns here, there is evidence that the skewing effect on the ethical debate we are warning about does happen. An example of this comes from George Winters sharing ‘his views on NIPT and how it influences decision-making’ [56, p. 14]. In citing the report by Bowman-Smart et al., Winter seems to have uncritically picked up the most prominent information included in the abstract, as well as statistical results as given in the relevant figures of the report without any further clarification. Particularly, Winter notes that:

[i]n an Australian survey of 235 women who had undertaken NIPT, Bowman-Smart et al. (2018) investigated views on existing and possible future NIPT, finding that 99.1% supported NIPT for Down syndrome screening, with 42.9% reporting they would consider abortion following a diagnosis of the condition [56, p. 14].

What is missing from this quote is, as we have argued above, the private context of testing undertaken by the women who took part in the survey, which may also be a factor likely to have shaped women’s responses about abortion. This should have had much more prominence in Bowman-Smart et al. and therefore in Winter because it is fundamental to understanding the data.

We argue that this case, and any others that follow, provide weight to our argument that unless authors highlight important information when presenting the findings of empirical research, there is a material risk that readers who retrieve those findings might not reproduce vital information. In other words, even unintentionally, if data is presented in this way it may encourage a distorted impression about the quality and the value of the primary research and its findings.

### Pesso et al. in Acharya and Ross

In their paper ‘Fragile X screening: attitudes of genetic health professionals’ Acharya and Ross cite Pesso et al. when they state that: ‘Despite these ethical concerns, the data that does exist show broad social acceptance of FrX prenatal testing among low-risk women in the US and other countries’ [57, p. 627]. Of course, if the readers of this paper also read the Pesso et al. paper in full, they would understand that the evidence that is suggested by Acharya and Ross for the broad social acceptance of FrX is *not* apparent.

In fact, Pesso et al. state that ‘… it is also likely that those who self-referred or were referred by a doctor are not representative of the population as a whole. Since women had to pay for the test themselves, a higher than average socio-economic group will have been screened’ [55, p. 613]. We argue that this is a good example of how a lack of clarity and full information about the findings of papers may unintentionally skew a debate. It is not that Pesso et al. have deliberately misled, but by not linking the limitations of a study clearly with the findings, there is a risk that others may use these findings in a way that is not representative of the actual findings and that the reader of this secondary work will not be, therefore, accurately informed.

### Does this all matter?

In this paper we have highlighted an issue that we recognise usually falls short of actual malpractice. It could even be argued that the issue here is not with the researchers but with those who do not carefully read the papers that present the researchers’ empirical data. However, we argue that it is important to the credibility of this kind of research that those generating and using empirical data in bioethics take *extra* care to highlight limitations, be *extra* transparent, and be *extra* reflexive. In addition to urging those who use empirical data to do so more critically and carefully, we argue there is an obligation on researchers reporting empirical work to acknowledge and attempt to control their own unconscious biases in the way that the data is reported. Until this is done, then unwanted secondary misuse of their research is very likely and the ethical debate — often in areas that have the potential to impact individual lives — will be unjustifiably skewed.

There has been a tendency for healthcare policy to be driven by technical and scientific discoveries; the history of prenatal screening is no exception [7]. Up until relatively recently, prenatal screening policies were driven not by women’s choice and autonomy, but by excitement over scientific discoveries [7]. Historically, these policies were often motivated by eugenic goals [7]. With the rise of the notion of respect for autonomy and the recognition that women’s choice is fundamental, there has been an attempt to reconcile prenatal screening practice with this notion of autonomous choice. It has been argued that — perhaps as a result of these different foundations — providing routine prenatal screening that is compatible with respecting the autonomy of women has proved problematic [7]. Whether the reader agrees with this analysis or not is a matter for another debate. However, what is clear is that the drivers of scientific innovation — the desire by many to screen out disability and significant commercial interests (e.g., the global NIPT market will reach USD 7.3 billion by 2024 [58]) — are powerful forces when it comes to the ethical debate around routine prenatal screening. While we know that empirical research is an important tool to help to illuminate ethical issues such as prenatal screening, if one wishes to do this work in research with the utmost integrity and accuracy, then awareness of the effects of bias – however, subtle — and guardedness against them must be a central concern for those engaged in these studies.

We, of course, have our own normative biases which, as part of the research in this paper we have reflected on and explored. Our own bias is, of course, a major reason for having shown interest in the paper by Bowman-Smart et al. and for having been able to identify the issues related to this report. While we accept that bias is a fact of life and its influence may not be conscious, we also argue that, just as measures are put in place to minimise the effect of bias in other human processes (such as recruitment and assessment), the same measures must be applied in academic journal articles which have the potential to significantly influence ethical debate and policy-making. An integrally important element of our own methodological approach to the philosophical bioethical method is the need to defend our work against counterarguments; that is, to consider our arguments from the opposing side of our position to ensure that the positions we take can be justified and defended against. Defending our ethical positions against counterarguments allows philosophical bioethical debate to guard against bias and avoid basing arguments only on intuition and thus to provide robust positions that will stand up to scrutiny. We suggest that this element of philosophical methodology should play a central role when bioethicists and others use empirical work to inform their conclusions. We suggest that best practice — both from a philosophical bioethics tradition and from a tradition around the methodology of the social sciences — requires the practice of reflexivity on the part of those who generate, use, and report empirical data that has an ethical application. Reflexivity is practiced by standing in the shoes of those with the opposite view(s) in order to identify and control the influence of bias in one’s work, being *extra* vigilant and *extra* cautious when it comes to engaging with empirical projects that have relevance to ethical issues.

Also, as we have acknowledged in the introduction, this takes a lot of effort and sometimes it might be impossible to recognise and control our biases. Thus, we argue that in addition to the researcher’s individual responsibility to protect their work from the influence of their own biases, there are still practical measures to consider which can be agreed upon on a collective level and can limit the impact of such biases as well as the risk of secondary misuse. We suggest that a secondary failsafe should be put in place; when a limitation of an empirical study reaches a certain level of seriousness in the manuscript (because the implications of the study being misread or misinterpreted through superficial reading are magnified), information about the limitation should not be confined to its standard placement in the “Limitations” paragraph in the Discussion but should also be highlighted in the abstract, if not the title. We call this idea a “Limitation prominence assessment” and suggest that it might become a new criterion to include in lists of considerations used by editors, reviewers, and submitting authors as they consider the quality of a manuscript's clarity. Under this criterion, those assessing a paper would be encouraged to evaluate the seriousness of the limitations of an empirical study and thus the risks of the study being misread or misinterpreted through superficial reading. If the limitations are serious and the risks are high, then serious consideration should be given to disclosing these limitations in the abstract (or title if possible) of any paper reporting on this study. In such cases, not representing these limitations in this way would require some justification before a paper could be accepted for publication.

## Conclusion

Empirical data has the potential to be hugely influential on researchers and policy makers when coming to a position on an ethical issue. It is, of course, important that all those working with empirical data in this area do so thoroughly, critically, and reflexively to ensure that research in this area maintains its integrity and accuracy. In this paper, we have argued that even when standard research protocols are followed, there can exist the danger that the way that data is presented may encourage others to miss vital nuances of the research undertaken, potentially skewing the debate in highly ethically, politically, and commercially sensitive areas. These issues are often very subtle and difficult to identify, particularly by those who share similar normative biases. As a result, one’s normative biases may risk obscuring the truth one seeks.

In this paper we have used practical examples from our own area of research, prenatal screening, to highlight this issue and to argue that awareness of this issue implies a further obligation on researchers when reporting their findings in ethically sensitive areas to be *extra* vigilant and *extra* cautious to minimise the potentially distorting effect of normative bias. Our own reflection on this issue has led us to suggest that it is crucial that we consider empirical data and research in bioethics from the viewpoint of those with opposing normative positions to guard against these issues. We recognise that even the best will in the world along with the practice of reading our work through the lens of those with oppositive normative views may not always be enough to illuminate these issues. Therefore, we recommend the implementation of a common rule, what we call the “Limitation prominence assessment”, that we propose for editors, reviewers, and submitting authors. These persons should be asked to consider the assessment when reviewing papers submitted for publication. This is one easily implementable and practical way to strengthen the integrity of this complicated intersection between bioethics, empirical research, and policy.

## Footnotes

1 We use the word ‘women’ here to represent the group of individuals who may be affected by routine prenatal screening. However, it is very important to recognise that there will be individuals who do not identify as women who may be pregnant or considering pregnancy. Thus, determining their views on this issue will also be very important.

2 To be clear, the word ‘intuition’ in this paper is used to indicate what we see as the common usage of this word to mean ‘an ability to understand or know something without needing to think about it or use reason to discover it, or a feeling that shows this ability’ (Definition of **intuition** from the Cambridge Academic Content Dictionary © Cambridge University Press [60]).
